# High-Cycle Fatigue Behaviour of Polyetheretherketone (PEEK) Produced by Additive Manufacturing

**DOI:** 10.3390/polym16010018

**Published:** 2023-12-20

**Authors:** Pedro Rendas, Alexandre Imperadeiro, Rui F. Martins, Bruno A. R. Soares

**Affiliations:** 1 UNIDEMI, Department of Mechanical and Industrial Engineering, NOVA School of Science and Technology, Universidade NOVA de Lisboa, 2829-516 Caparica, Portugalrfspm@fct.unl.pt (R.F.M.); ba.soares@fct.unl.pt (B.A.R.S.); 2 Laboratório Associado de Sistemas Inteligentes, 4800-058 Guimarães, Portugal

**Keywords:** Polyetheretherketone (PEEK), material extrusion, additive manufacturing, mechanical properties

## Abstract

Polyetheretherketone (PEEK) is the leading high-performance thermoplastic biomaterial that can be processed through material extrusion (ME) additive manufacturing (AM), also known as three-dimensional (3D) printing, for patient-specific load-bearing implant manufacture. Considering the importance of cyclic loading for load-bearing implant design, this work addresses the high-cycle fatigue behaviour of 3D-printed PEEK. In this work, printed PEEK specimens are cyclically loaded under stress-controlled tension–tension using different stress levels between 75% and 95% of printed PEEK’s tensile strength. The experimental results are used to document 3D-printed PEEK’s fatigue behaviour using Basquin’s power law, which was compared with previous fatigue research on bulk PEEK and other 3D-printing materials. As a pioneering study on its fatigue behaviour, the results from this work show that 3D-printed PEEK exhibits an above-average fatigue strength of 65 MPa, corresponding to about 75% of its tensile strength. Fracture surface analysis suggests that a transition can occur from ductile to brittle fracture with maximum stresses between 85% and 95% of the tensile strength. Evidence of crack propagation features on fracture surfaces under scanning electron microscope (SEM) observation suggests crack initiation in void defects created by printing deposition that propagates longitudinally through line bonding interfaces along layers. Considering this, 3D-printed PEEK’s fatigue behaviour can be strongly related to printing conditions. Further research on the fatigue behaviour of 3D-printed PEEK is necessary to support its use in load-bearing implant applications.

## 1. Introduction

In the fields of engineering and manufacturing technologies, additive manufacturing (AM), also known as three-dimensional (3D) printing, has been one of the main subjects of recent research. The significance of AM comes from its ability to manufacture complex geometry components while reducing material waste when compared to conventional subtractive manufacturing [1,2]. With AM, components are built layer by layer based on computer-aided design (CAD) models, allowing for greater design flexibility and making it especially appealing for customised design applications. Considering this, recent advances in the reliability of AM technology and materials have made these manufacturing techniques highly suitable for demanding applications in the aerospace and medical industries [3]. In the medical field, in particular, AM-compatible high-performance biomaterials can be used in the manufacture of patient-specific medical devices designed based on 3D models obtained from medical imaging or even in the manufacture of complex scaffold structures for tissue engineering [4,5,6].

Within the category of biomaterials processable through AM, polyetheretherketone (PEEK) is a high-performance thermoplastic material that has the potential to replace metals in orthopaedic, trauma, and spinal treatment applications. This potential originates from PEEK’s high strength-to-weight ratio and chemical stability, which make it biocompatible, resistant to in vivo degradation, and thus suitable for load-bearing implant manufacture [7,8,9]. Furthermore, PEEK’s rigidity and strength are closer to the properties of human bone than those of metals and thus can reduce the stress shielding of the treated bone. Stress shielding has been shown to cause bone resorption [10] and consequently result in poor implant performance. As a thermoplastic, PEEK can be processed using material extrusion (ME) AM, also known as fused deposition modelling (FDM^®^, Stratasys, USA) or fused filament fabrication (FFF), where filament material is deposited in its molten state through a moving nozzle. Considering its potential, this technique has been used in previous research to document 3D-printed PEEK’s performance in different load-bearing implant device applications like intervertebral cages [11,12] and cranial plates [13].

In the context of medical implant manufacturing, the utilisation of 3D-printed PEEK, while promising, remains challenging considering these applications’ mechanical performance and bioactivity requirements [14]. The mechanical behaviour of 3D-printed PEEK is strongly associated with printing parameters. For instance, FFF, commonly referred to as 3D printing, involves thermal processing that influences PEEK’s crystalline content and is related to PEEK’s mechanical properties. In this thermal processing, slower cooling rates produce higher crystallinity PEEK [15] that displays higher strength [16]. Considering this, printing temperatures can be adjusted for slower cooldown rates to reduce warping defects and increase crystallinity, resulting in 3D-printed PEEK with higher tensile strength and elastic modulus [17,18].

In addition to crystallinity, improved bonding conditions can also enhance 3D-printed PEEK’s mechanical behaviour. These bonding conditions have been related to process temperatures and deposition strategy using heat transfer models where higher temperatures and slower cooldown rates can increase polymer chain mobility and fluidity upon deposition [19,20,21]. The increased fluidity upon deposition with higher nozzle temperatures results in larger adhesion surfaces and increases both density and strength [22,23]. These adhesion surfaces play an important role in the mechanical behaviour of 3D-printed PEEK. Weak molecular cross-linking in these interfaces makes printed components anisotropic in the sense that loads carried axially through the lines will be supported better than those causing shear where the filament is bonded [24,25,26,27]. This means that the build orientations where deposition is aligned with loading can significantly improve the mechanical behaviour of 3D-printed PEEK [28,29]. Additionally, adhesion surfaces are also related to the void contents of printed components. The infill parameter is directly related to the effective cross-section area for load support, and maximum infill should be used to increase the strength of PEEK prints. However, even for 100% infill, the void contents of PEEK prints can account for as much as 8% of the volume [30]. In sum, research makes clear that the mechanical behaviour of 3D-printed PEEK is linked not only to process parametrization but also to load configuration.

With its potential for the manufacture of load-bearing medical devices, understanding PEEK’s mechanical behaviour under different load conditions is an essential step to fulfilling the requirements for these applications. Since human movement can create cyclic musculoskeletal loading that can reach millions of cycles per year [31], fatigue behaviour is one of the most important considerations in the field of biomaterials [32]. While most research is focused on documenting its behaviour under monotonic load conditions, research addressing the fatigue behaviour of PEEK is scarce. The fatigue behaviour of bulk PEEK has been shown to outperform other polymers due to its semi-crystalline nature, where molecular cross-linking allows for higher fatigue strength [33,34]. Like other thermoplastic materials, PEEK’s fatigue life is significantly related to the configuration of the cyclic loading since test frequency and amplitude can create a thermally dominant regime where fatigue life is reduced significantly [35,36]. PEEK’s fatigue behaviour is also significantly different under stress-controlled and strain-controlled conditions [37]. Considering this and given the substantial number of loading cycles inherent to implant applications, the focus of research on biomaterials such as PEEK should be guided towards their high-cycle fatigue under stress-controlled conditions.

The high-cycle tension–tension fatigue behaviour of bulk PEEK has been documented in comparison to surface porous PEEK [38] and PEEK-based composites like carbon fibre-reinforced PEEK (CF-PEEK) [39,40] and PEEK loaded with hydroxyapatite (PEEK-HA) [41,42,43]. In these works, bulk PEEK samples are reported to withstand infinite life with maximum stresses of up to 75% of their tensile strength. However, the introduction of either filler materials or porosities into neat PEEK generally reduces fatigue strength due to crack nucleation in the PEEK-filler interface. On the other hand, the presence of higher contents of fibres in PEEK’s matrix can also oppose crack propagation [44,45]. All this suggests that the presence of voids and anisotropy from the deposition of 3D-printed PEEK can have significant effects on its high-cycle fatigue behaviour that need to be addressed.

In this work, the high-cycle fatigue behaviour of 3D-printed PEEK is documented. PEEK specimens were printed using optimal parameters obtained in previous works [46] and tested under tension–tension cyclic loading with different stress levels concerning the tensile strength of printed specimens. With this, the S-N (Stress-Life/Number of cycles) curve fitting the experimental data is plotted to document 3D-printed PEEK’s fatigue behaviour, and the fracture surfaces of specimens that failed under different stress levels were analysed to better understand the failure mechanisms involved. Additionally, results from previous works are used to assess how printed PEEK’s fatigue behaviour compares to other 3D printing materials. To the authors’ knowledge, this is the first work reporting on the high-cycle fatigue of 3D-printed PEEK, where the results further support its potential as the leading thermoplastic biomaterial for high-performance AM applications like load-bearing implant manufacture.

## 2. Materials and Methods

### 2.1. Materials and Equipment

For the fatigue experiments, tensile specimens were printed with neat PEEK filament (Apium PEEK 450 Natural, Apium Additive Technologies GmbH, Karlsruhe, Germany) with a diameter of 1.75 mm. Some material properties specified by the manufacturer for this filament are included in Table 1. The test specimens were printed with a 0.4-mm nozzle equipped on an Apium P220 printer (Apium Additive Technologies GmbH, Karlsruhe, Germany), which is designed for high-performance printing of high-temperature materials such as PEEK. Prior to specimen printing, the PEEK filament was dried in the Apium F300 filament dryer (Apium Additive Technologies GmbH, Karlsruhe, Germany) for 4 h at 120 °C and then maintained at 60 °C for conditioning and printing.

### 2.2. Specimen 3D Printing

**Figure 1:**
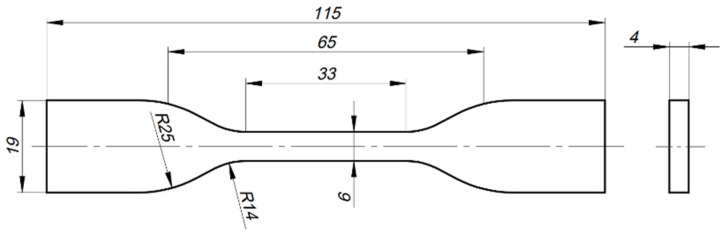
ASTM D638 tensile specimen type IV for the fatigue tests. Dimensions are in millimetres.

**Figure 2:**
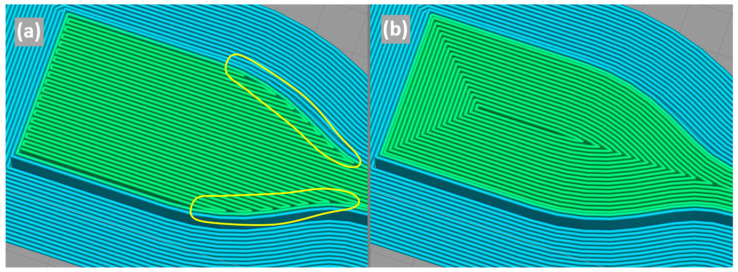
Deposition pattern discontinuities in the cross-section reduction area of the specimen with the rectilinear pattern (**a**) and with the concentric pattern (**b**).

Tensile test specimens were designed and dimensioned following the specifications from ASTM D638 [48] for specimen type IV, as illustrated in Figure 1. The G-code printing file was generated using the software Simplify3D (v4.1.2, Simplify3D, Blue Ash, OH, USA) with the specimen built flat on the print bed, and the printing parameters were selected based on parameter optimisation results for tensile strength from a previous work [46]. Additionally, to avoid discontinuities in the cross-section reduction area of the specimen with the rectilinear deposition (Figure 2a), the concentric deposition strategy was used, which is a pattern that also aligns the deposited lines with the specimen tensile loading (Figure 2b).

For the printing temperatures, the specimens were printed with a nozzle temperature of 485 °C, a print bed temperature of 130 °C, and a zone heater temperature of 130 °C. Zone heater temperature refers to the printer’s adaptive heating system surrounding the nozzle, which lowers cooldown rates and increases print quality. This temperature contributes to deposition zone preheating, which is related to the crystallinity and mechanical properties of 3D-printed PEEK [49]. Also, following the parameter optimization results, layer height was set to 0.2 mm and line width was set by the slicer to 0.48 mm, given the nozzle diameter of 0.4 mm. The default printing speed was set to 2000 mm/min with underspeed factors of 40% for 2 perimeter lines, 80% for solid infill, and 40% for the first layer, while the non-printing movement speed was set to 4800 mm/min. Lastly, a large brim of 25 concentric-type laps was added to prevent detachment from the print bed. Table 2 provides a summary of the printing parameters used for the PEEK tensile specimens.

### 2.3. Fatigue Testing

The high-cycle fatigue behaviour of 3D-printed PEEK was tested following the guidelines of ASTM’s standard D7791-12 [50] for the uniaxial fatigue properties of plastic materials. For these tests, a cyclic sinusoidal tension–tension load with *R* = 0.2 (R = $\sigma_{min}$/$\sigma_{max}$) was applied to the tensile specimens using the servo-hydraulic universal testing machine MTS 312.31 (MTS Systems Corp., Eden Prairie, MN, USA) with a 100 kN load cell. If 10⁶ cycles are reached, the test is stopped, and infinite fatigue life is considered (runout). Additionally, the temperature of the test specimens was monitored using an infrared (IR) thermal imaging camera (Fluke Ti400, Fluke Corporation, Everett, WA, USA), for which measurement parameters were adjusted with ambient temperature readings of the specimens.

**Figure 3:**
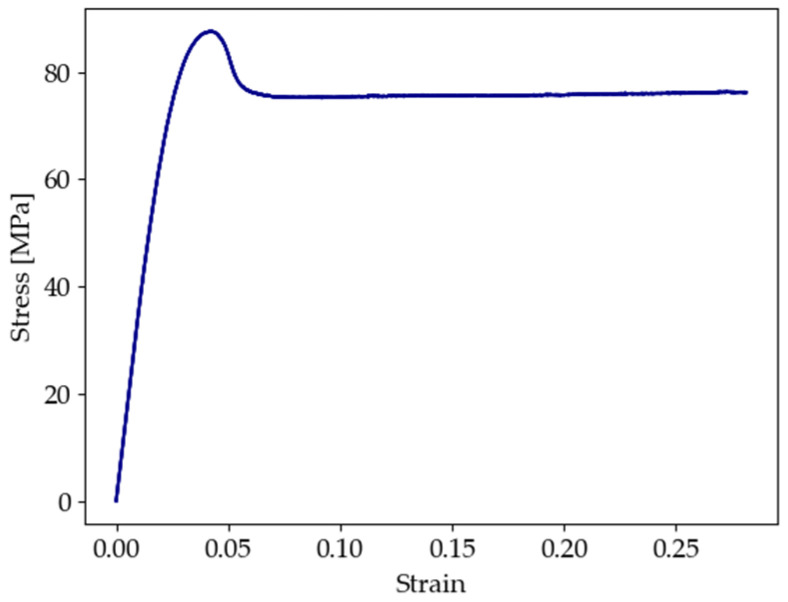
Engineering stress–strain curve from the monotonic tensile testing of 3D-printed PEEK [46].

Fatigue tests were performed on the 3D-printed PEEK specimens under load-controlled conditions for three different stress levels. For each level, the maximum stress applied ($\sigma_{max}$) corresponded to 75%, 85%, and 95% of the tensile strength of 86.7 MPa that was previously determined using similar 3D-printed PEEK specimens [46]. Moreover, a representative engineering stress–strain curve obtained in a monotonic tensile test is provided in Figure 3 [46], where it can be seen that the tensile strength is almost coincident with the yield stress for PEEK, as in accordance with the ASTM D638 standard. Hence, considering a stress ratio (R) of 0.2, the stress amplitude ($\sigma_{amp}$) for the fatigue tests was calculated using Equation (1):

(1) $$\sigma_{amp}=0.4\;\sigma_{max}$$

To test each specimen, both the maximum and minimum loads (F) were calculated given the stress (σ) level and the specimen’s measured gauge cross-sectional area ($A_{cs})$ according to Equation (2). These load values were then used to calculate both load amplitude and mean load to programme the testing machine. The values for the stresses and loads calculated for each test specimen are provided in Table 3. The results from the fatigue tests were plotted with the stress amplitude ($\sigma_{amp}$) versus the number of cycles to failure ($N_{f}$) on a logarithmic scale, and Basquin’s model was used to calculate the best-fit curve according to Equation (3), where both A and b are constants.

(2) $$F=\sigma \cdot A_{cs}$$

(3) $$\sigma_{amp}=A\cdot N_{f}^{b}$$

### 2.4. Fractography

The fracture surfaces of the experimental fatigue-tested specimens were observed and analysed both using a high-resolution camera (ISM-PM200sb, Insize Co., Suzhou, China) and a scanning electron microscope (SEM) (model SU3800, Hitachi Ltd., Tokyo, Japan). The SEM micrographs of the fracture surfaces of PEEK specimens were taken using a low vacuum mode for non-conductive materials at 30 Pa of pressure and with an accelerating voltage of 20 kV. Using this mode, images were taken at relatively low magnifications between ×130 and ×500 and using the detection of backscatter electrons for 3D surfaces (BSE-3D) to observe the different regions of the fracture surfaces of the tested specimens.

## 3. Results and Discussion

### 3.1. High-Cycle Fatigue of 3D-Printed PEEK

**Figure 4:**
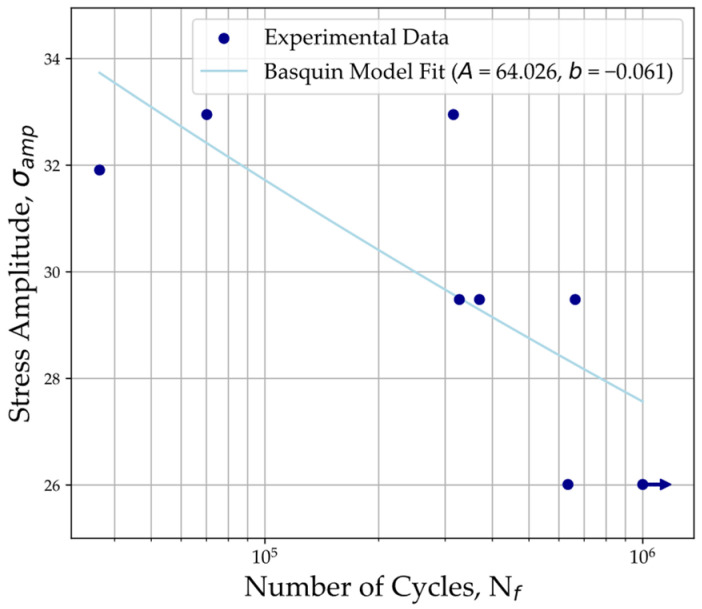
S-N plot of experimental data with Basquin’s model fit (A=64.026; b=−0.061). Correlation coefficient: 0.543. Arrow indicates runout.

**Figure 5:**
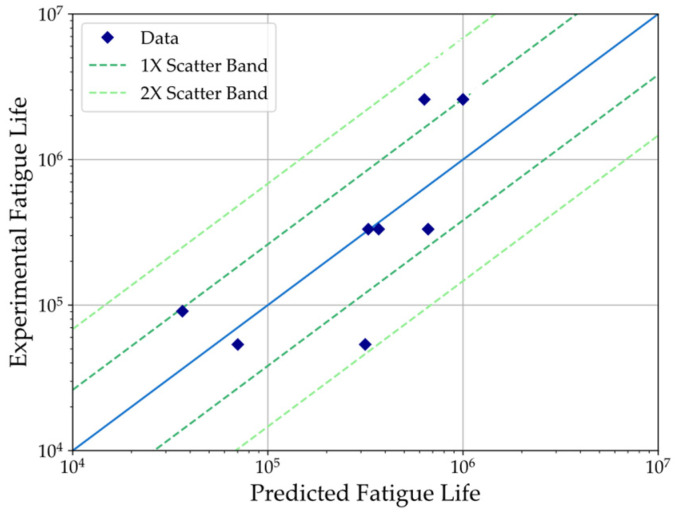
Experimental fatigue life versus predicted fatigue life with Basquin’s model fit.

The results from the fatigue tests performed on 3D-printed PEEK tensile specimens are provided in Table 4 and plotted using a semi-logarithmic scale in Figure 4 along with the Basquin’s model best-fit curve. The values obtained for the S-N curve’s constants *A* and *b* correspond to 64.026 and −0.061, respectively, and are also provided in the legend of the plot in Figure 4. Using this model, the predicted fatigue life is plotted with the experimental fatigue life in Figure 5 to assess the correlation between predictions based on the S-N curve and the experimental results. The plotted data fit within the scatter band for one standard deviation error of prediction, except for specimens 2 and 7.

**Figure 6:**
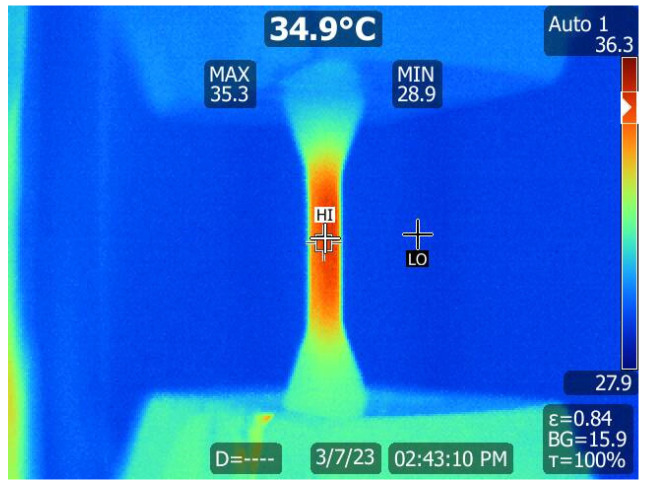
Specimen surface peak temperature reading with an IR camera.

At the beginning of each test, the temperature of the gauge section of the tensile specimens increased by about 10 °C for all stress levels and stabilised at around 35 °C (Figure 6). This increase in temperature is expected with cyclic-loading polymers since elastic deformation energy is dissipated as heat [37]. For higher cyclic maximum stresses, the fatigue behaviour of PEEK can become thermally dominant due to hysteretic heating, which significantly reduces fatigue life [35]. In this work, the temperature of all tested specimens stabilised under 15,000 cycles without any significant temperature variation for the remainder of the fatigue test. The measured temperature variations are similar to the variations previously reported for the mechanically dominated fatigue regime of PEEK [35,36], where specimens under this regime withstood more than 10⁴ cycles.

As these results show, the fatigue life of the tested specimens varied significantly within the same testing conditions for stress amplitude and mean stress. In the fatigue tests conducted with maximum stress in the range of 92–95% of 3D-printed PEEK’s tensile strength, the total number of cycles to failure for specimen number 5 was about 10 times higher than the number of cycles for specimen 4. These large-scale variations are normal in fatigue life and can be caused by the differences in the specimens’ mesostructure, which are typical of FFF deposition and can result in different configurations of void defects even for the same printing conditions. The presence of void defects in 3D-printed PEEK is largely unavoidable, even for 100% infill prints, and it also depends on printing conditions. Although the printing parameters used for the specimens were based on the optimisation results from previous works [46], there can still be differences in the void defect configuration of the tensile specimens printed for this work. With this, the effects of these differences seem more significant in the fatigue tests with higher mean stresses and stress amplitudes, as is the case for specimens 4 and 5, as mentioned above.

Apart from this, the average fatigue life of 3D-printed PEEK under each stress condition decreased with the increase in stress level, as expected. As the maximum stress was decreased to 75% of 3D-printed PEEK’s tensile strength, infinite fatigue life (runout) was reached (specimen 8), which corresponds to a fatigue strength of 65 MPa. This stress level for the fatigue strength of 3D-printed PEEK is similar to the stress level of 70–75% at the endurance limit reported for bulk PEEK specimens [33,39,40,42,44]. The experimental results show that the fatigue behaviour of 3D-printed PEEK can be compared to that of bulk extruded or moulded PEEK specimens.

**Figure 7:**
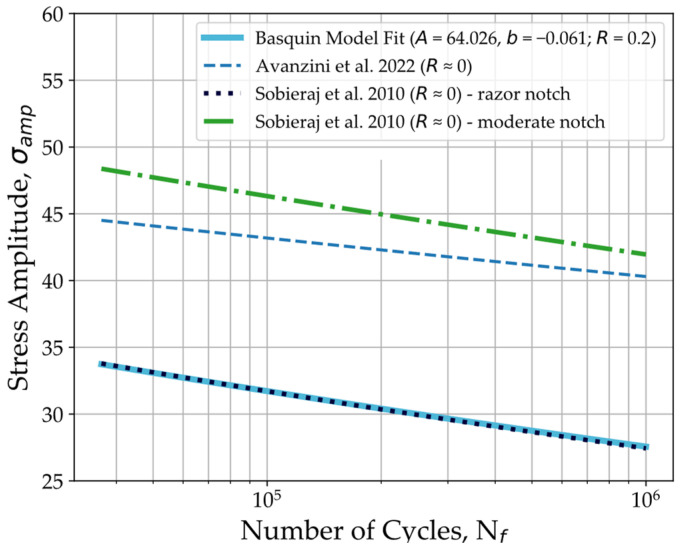
S-N curve comparison to research results for bulk PEEK [40] and notched PEEK specimens [51].

Figure 7 plots the S-N curve obtained in this work for 3D-printed PEEK along with curves obtained in previous works where neat PEEK specimens were cyclically loaded under tension–tension conditions [40,51]. The plotted S-N curves were obtained from neat PEEK specimens by Avanzini et al. [40] and from differently notched PEEK specimens aged in PBS at 37 °C by Sobieraj et al. [51], in both cases machined from extruded PEEK. The fatigue strength reported by Avanzini et al. [40] of 75 MPa corresponds to about 75% of PEEK’s ultimate tensile strength (UTS), which is the same stress level as the fatigue strength reported in this work. Considering this, the fatigue behaviour of 3D-printed PEEK resembles that of bulk PEEK when accounting for the lower tensile strength of the 3D-printed specimens. Similarly, compared to the results of Sobieraj et al. [51], 3D-printed PEEK’s fatigue behaviour is analogous to the behaviour of the razor-notched PEEK specimens. In this work, different notch dimensions are tested with a stress intensity factor of 2.1 for the specimens with a moderate notch with 0.9 mm of radius and a stress intensity factor greater than 10 for the razor-notched specimens. With the razor-notched specimens, the higher stress intensity factor corresponded to a decrease in stress amplitude and maximum stress for the same fatigue life. This is represented by a shift downward of the S-N curve from specimens with moderate notches to specimens with razor notches. The fatigue behaviour of 3D-printed PEEK seems equivalent to notched PEEK with a high stress intensity factor, which can be explained by the presence of void defects in the tensile specimens created by printing deposition. This relation between the void contents and the fatigue behaviour suggests the fatigue strength of 3D-printed PEEK can be improved by using printing strategies that reduce the volume and size of these void defects, like interlayer line offset, as presented in previous works [46].

These relations between the fatigue behaviour of 3D-printed and bulk specimens are similar to those demonstrated in previous works for polylactic acid (PLA) [52]. To the authors’ knowledge, there is no previous research documenting the fatigue behaviour of 3D-printed PEEK. However, previous studies have conducted fatigue experiments on specimens 3D-printed with different polymers. This research has shown that printing parameters like printing temperatures, layer height, nozzle diameter, and infill can significantly affect the fatigue behaviour of 3D-printed specimens [53,54,55]. Despite this, the printing parameters that can influence the fatigue behaviour the most appear to be those related to the load alignment with deposition, like raster angle and build orientation. As is the case for monotonic loading conditions, loads carried axially through the deposited lines are supported better than those causing shear where the filament is bonded [25]. For these reasons, research has shown that fatigue life is extended when build orientation places layers longitudinally [56,57,58] and raster angles with lines parallel to the load direction [53,59,60]. This configuration corresponds to the printing orientation and raster angle chosen to print the PEEK specimens in the present work.

**Figure 8:**
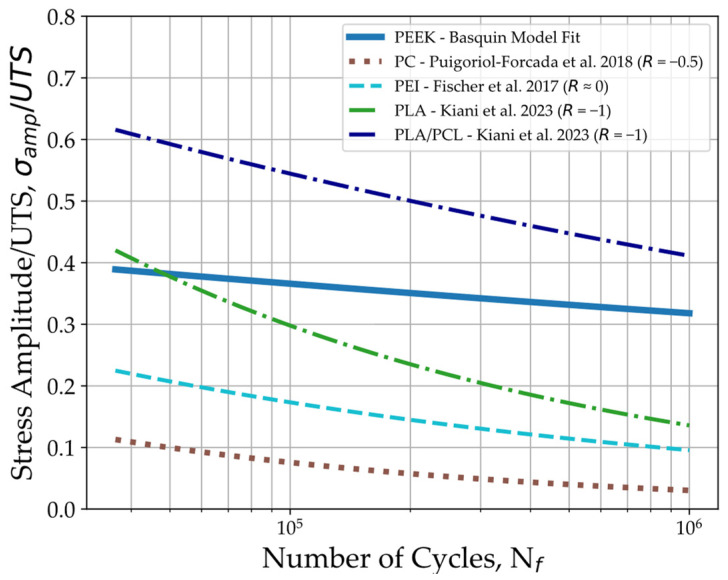
S-N curve comparison to 3D-printed PC [56], PEI [57], and both neat PLA and PLA/PCL blends [61].

To evaluate how the fatigue behaviour of 3D-printed PEEK compares to other 3D-printed materials, the S-N curve obtained in this work is plotted in Figure 8 along with the S-N curves for 3D-printed polycarbonate (PC) [56], PEI [57], PLA, and PLA/PCL [61] with the stress amplitude normalised to the respective material’s UTS. The results plotted from these works consider the build orientation where the specimens lay flat on the print bed, which is equivalent to the orientation used in this work. Despite some differences in test conditions, Figure 8 suggests that 3D-printed PEEK’s fatigue endurance is less sensitive to stress amplitude and mean stress increases compared to other materials. In addition to PEEK’s higher strength and stiffness, printed PEEK seems to withstand higher stresses under fatigue loading when compared to other high-performance polymers like PC and PEI, even in relation to their respective tensile strengths.

Compared with PLA, the normalised stress of PEEK at the endurance limit is approximately double that of PLA, despite both materials exhibiting similar fatigue life under higher normalised stress amplitudes. Interestingly, the introduction of a non-rigid polymer like PCL to the PLA filament material helped attenuate fatigue damage and improved the fatigue strength of the 3D-printed specimens [61]. Nevertheless, this increase was obtained at the expense of the material’s tensile strength. One of the reasons suggested in this work for this improvement in fatigue behaviour is the increase in PLA’s crystallinity percentage with the addition of PCL.

Nevertheless, the comparisons displayed in Figure 7 and Figure 8 refer to fatigue experiments conducted under different stress ratios that can significantly alter the specimens’ fatigue response. In Figure 7, the S-N curves for the bulk PEEK specimens were obtained with a stress ratio of R=0, while in Figure 8, the plotted curves for 3D-printed polymer materials were obtained with different stress ratios that include fully reversed loading with R=−1. To enable comparison, a mean stress correction based on the Goodman relation [62] was applied to calculate the fatigue strength for R=−1 ($\sigma_{f(R=-1)})$ using the stress amplitude $\left(\sigma_{f}\right)$, mean stress ${(\sigma }_{m})$ at the runout of 10⁶ cycles, and tensile strength ($\sigma_{u})$ of the specimens through Equation (4):

(4) $$\frac{\sigma_{f}}{\sigma_{f(R=-1)}}=1-\frac{\sigma_{m}}{\sigma_{u}}$$

The fatigue strength values with the mean stress correction for the results plotted in Figure 7 and Figure 8 are provided in Table 5. In these works, if fatigue strength was not available, the Basquin equation was used to estimate the stress amplitude at runout. As expected, the Goodman relation (Equation (4)) implies that fatigue strength is lower with higher mean stresses, meaning that higher stress ratios result in a shift downward of the fatigue curve in the S-N plot. The comparison of results can be seen in Table 5, where 3D-printed PEEK’s stress amplitude at runout increases from 65% of the stress amplitude presented by Avanzini et al. [40] for bulk PEEK to about 72% of its stress amplitude.

Finally, PEEK’s semi-crystalline nature can be an important factor in its fatigue resistance, considering that the extension of crystalline molecules helps dissipate energy from cyclic loading and hinders crack initiation [33,34]. Moreover, the crystallinity of 3D-printed PEEK is influenced by cooling conditions [14]; hence, the fatigue behaviour of PEEK can also be related to the thermal processing history of the printing process. The relatively high crystallinity of 33.5% obtained with the printing parameters used in this work [46] can be one of the reasons why 3D-printed PEEK displays high fatigue resistance compared to other 3D printing materials. Furthermore, PEEK also displays an above-average strength, which results in a significantly higher fatigue strength of 65 MPa when compared to other materials, further supporting its use in high-performance applications that benefit from the advantages of additive manufacture.

### 3.2. Fracture Surface Analysis

The fracture surfaces of the specimens that failed under different stress levels were examined both using a high-resolution camera and SEM. With this, images of the fracture surfaces can provide additional insight into the fracture mechanics involved in the fatigue failure of 3D-printed PEEK and how these can be related to the mesostructure that comes with FFF deposition.

**Figure 9:**
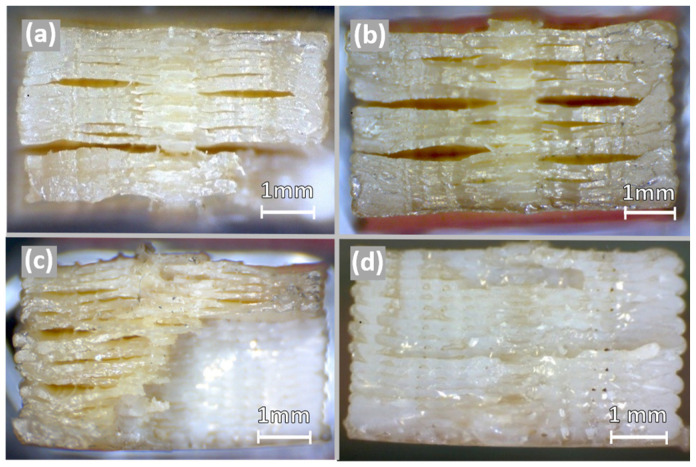
Fracture surface macrographs of specimen 7 tested with a stress level of 75% (**a**), specimen 1 tested with a stress level of 85% (**b**), and specimen 5 (**c**) and 6 both tested with a stress level of 95% (**d**).

The fracture surfaces of specimens 7, 1, 5, and 6 are compared in Figure 9, where a distinction can be seen between the lower 75% and 85% stress levels endured by specimens 7 and 1 (Figure 9a,b) and the 95% stress level of specimen 6 (Figure 9d). In this comparison, stress whitening can be seen in the fracture surface of specimen 6, which is subjected to a higher stress level, while specimen 1 shows a glassy centre region and evident signs of delamination. With the lower stress levels of specimens 7 and 1, delamination can be caused by the separate yielding of regions that bond differently during the printing process. This yielding can mean that localised plastic deformation occurred prior to failure, thus increasing heating and causing ductile fracture. Additionally, the fracture surface of specimen 1 also shows that delamination occurs at layer interfaces and stops near the centre region, which has a slight over-extrusion caused by the concentric deposition. This over-extrusion, despite resulting in a denser and stronger region, can also present larger-scale void defects [46] that can act as crack nucleation sites in fatigue loading.

Regarding the image of specimen 6 (Figure 9d), subject to the higher stress level, fracture appears to occur almost in a single plane transverse to the fatigue loading. With the discontinuities in the mesostructure of 3D-printed specimens, a single-plane fatigue fracture suggests that there was almost no plastic deformation and that crack propagation occurred very fast. Considering this, the differences in the fracture surface of specimen 1 and specimen 6 suggest that a transition from ductile to brittle fatigue fracture occurs as the stress level is increased, which is in line with the results from previous works on bulk PEEK [40,63]. This transition can be explained by the higher strain rate, which is applied when the stress level is increased for the same test frequency, since PEEK displays higher tensile stiffness and lower elongation at break when the strain rate is increased [64]. Despite this, as the fracture surface of specimen 5 shows (Figure 9c), the two described regions can both be present, indicating that the fatigue failure of PEEK can involve different mechanisms that are dependent not only on the stress level applied but also on printing outcomes.

**Figure 10:**
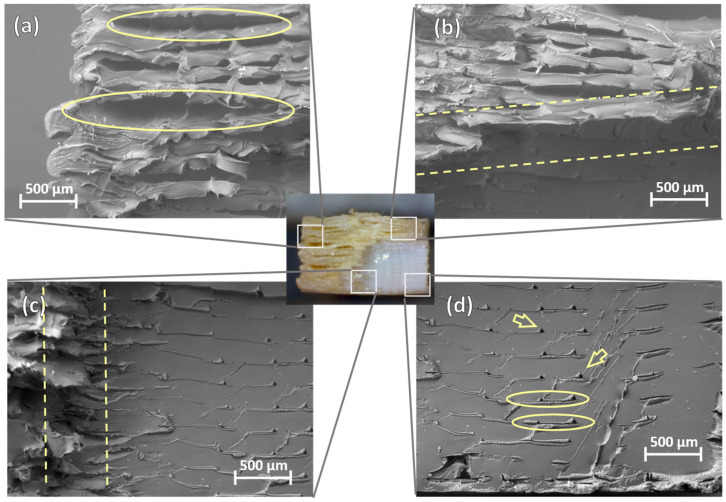
SEM micrographs of specimen 5, loaded with a stress level of 95%, displaying the plastic deformation (**a**), transition (**b**,**c**), and flat planar (**d**) of fracture surface regions.

In addition to macroscopic images, which can be limited in detail, SEM observation of these fracture surfaces provides additional information on the fracture mechanisms involved in the fatigue failure of 3D-printed PEEK. For the higher stress level of 95% of printed PEEK’s tensile strength, the fracture surface of specimen 5 is displayed in Figure 10, showing both regions described previously. In the glassy region, there is clear evidence of plastic deformation and consequent separation of weakly bonded regions from the printing process, as shown in the highlighted regions of Figure 10a. This separation occurs mainly between the deposited layers and even avoids smaller bonding interfaces between lines of the same layer. This weaker bonding between layers is caused by the larger temperature difference between the printed material and the deposition zone. The ductile fracture region transitions to the smooth region through these weak interfaces between layers, as marked in Figure 10b, and through the interface of the stronger over-extruded midsection mentioned above, as Figure 10c highlights. Conversely, Figure 10d shows the smooth fracture surface region, where void defects created between deposited lines are indicated by the arrows while slight layer delamination is indicated by the contouring.

**Figure 11:**
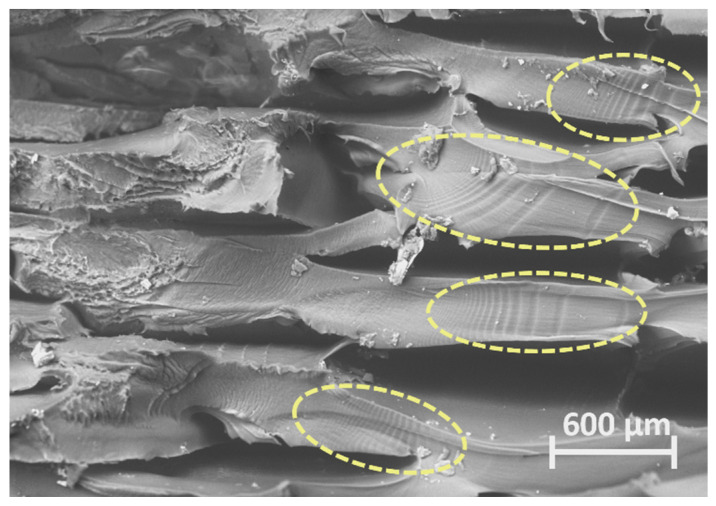
SEM micrograph of striations in the fracture surface of specimen 5 tested with a stress level of 95%.

In the smooth and planar region of the fracture surface, there is no clear evidence or markings of crack propagation. However, the region of Figure 10b shows striation bands under higher magnification, as contoured in Figure 11, where the distance between each band corresponds to crack growth per cycle. According to these markings, crack nucleation appears to have started in the centre region of the specimen and propagated horizontally through lines of the same layer. Crack nucleation in the centre region can be attributed to the larger-scale void defects resultant of over-extrusion, as previously reported for 3D-printed PEEK [46]. Considering this, the analysis of the fracture surface of specimen 5, loaded with the highest stress level, suggests that the smooth and planar brittle fracture region failed under high crack propagation rates.

**Figure 12:**
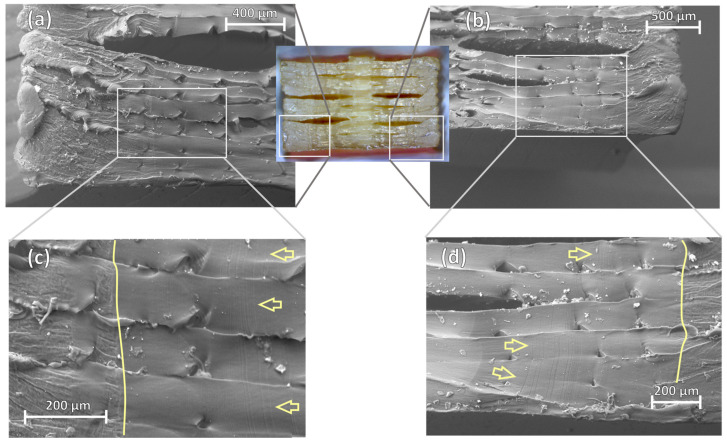
SEM micrographs of the left (**a**,**c**) and right (**b**,**d**) sections of the fracture surface of specimen 1 tested with a stress level of 85% where opposite crack propagation features are identified originating from the centre region.

As for the lower stress level of 85% of the specimen’s tensile strength, Figure 12 also presents the separation of layer interfaces (Figure 12a,b), but in this case, the fracture surface appears smoother and flatter in the areas surrounding these delaminations. At higher magnifications, it is also possible to see fatigue striations marked by the arrows that seem to radiate from the centre region of the specimen (Figure 12c,d). Following this direction for crack propagation, the smooth appearance of the fracture surface transitions to a roughened texture in the perimeter section of the specimen. This transition is highlighted by the contouring and can correspond to an instantaneous fracture due to overloading. These features suggest that the crack was initiated in a larger-scale void defect formed by the over-extrusion of the mid-section of the specimen, which propagated horizontally and outwards through line bonding interfaces. This means that the fatigue life of 3D-printed PEEK could be strongly associated with the void contents of printed specimens and, therefore, can be related to the deposition stability on 100% infill-dense prints.

## 4. Conclusions

This work presents a pioneering investigation of the high-cycle fatigue behaviour of 3D-printed PEEK. For this, fatigue experiments were conducted on 3D-printed specimens subject to stress-controlled tension–tension cyclic loading at varying stress levels relative to printed PEEK’s tensile strength.

From our experiments, S-N curve parameters were derived based on Basquin’s power law equation, which corresponds to a fatigue strength coefficient (*A*) of approximately 64.026 and a fatigue strength exponent (*b*) of approximately −0.061. Additionally, 3D-printed PEEK exhibited a fatigue strength of 65 MPa, equivalent to about 75% of printed PEEK’s tensile strength, which is consistent with the results from previous research on bulk PEEK samples.

The S-N behaviour of printed PEEK was compared to the results of previous research, where 3D-printed PEEK’s fatigue behaviour was associated with the behaviour of notched PEEK with a high stress intensity factor. Nevertheless, when compared with other 3D printing materials, PEEK shows above-average fatigue strength, especially under extended fatigue life. This is a promising result for applications that demand fatigue resistance, such as patient-specific load-bearing implant manufacturing.

Fracture surface analysis revealed a possible transition from ductile to brittle fracture between the cyclic loading at 85% and 95% of printed PEEK’s tensile strength. Furthermore, crack propagation features observed using SEM suggest that cracks initiate in void defects from printing deposition, which propagate through line bonding interfaces of the same layer. This emphasises the need for quality control and defect minimization during the 3D printing process to enhance printed PEEK’s fatigue resistance.

Given the potential of 3D-printed PEEK for load-bearing implants, additional research is essential to understand the relationships between process parameters and its high-cycle fatigue behaviour under different load conditions. Moreover, the mechanical behaviour of 3D-printed PEEK under cyclic loading conditions can be addressed in collaboration with medical experts for patient-specific implant design applications like the treatment of cranial and maxillofacial trauma, where the use of 3D-printed PEEK presents great potential.

## Figures and Tables

**Table 1:** Apium PEEK 450 Natural filament properties [47].

Filament Material Properties	
Density, *ρ* [g/cm^3^]	1.3
Elastic modulus, *E* [GPa]	4.0
$\mathrm{Tensile}\;\mathrm{strength},\;\sigma_{m}$ [MPa]	98
$\mathrm{Tensile}\;\mathrm{elongation},\;\varepsilon_{m}$ [%]	45
$\mathrm{Glass}\;\mathrm{transition}\;\mathrm{temperature},\;T_{g}$ [°C]	143
$\mathrm{Melting}\;\mathrm{temperature},\;T_{m}$ [°C]	343

**Table 2:** Tensile specimen printing parameters set in Simplify3D.

FFF Printing Parameters				
Nozzle temperature	485 °C	Layer height		0.20 mm
Bed temperature	130 °C	Extrusion width		0.48 mm
Zone heater temperature	130 °C	Printing speed		2000 mm/min
Deposition pattern	Concentric	Underspeed	Outline	40%
Deposition sequence	Inside-Out		Solid infill	80%
Perimeter shells	2		First layer	40%
Brim outlines	25	X/Y movement speed		4800 mm/min

**Table 3:** Loading parameters for the fatigue tests of the tensile specimens under different stress levels.

Stress Level	$\sigma_{max}$ [MPa]	$\sigma_{mean}$ [MPa]	$\sigma_{amp}$ [MPa]	Specimen Number (#)	$A_{cs}$ [mm^2^]	$F_{mean}$ [N]	$F_{amp}$ [N]
75%	65.0	39.0	26.0	7	26.43	1030	687
				8	26.52	1030	690
85%	73.7	44.2	29.5	1	26.45	1170	780
				2	26.29	1160	775
				3	26.67	1180	786
92%	79.8	47.9	31.9	4	27.82	1330	888
95%	82.4	49.5	32.9	5	25.97	1280	856
				6	24.96	1230	822

**Table 4:** Fatigue test results.

Specimen Number	$\sigma_{mean}$ [MPa]	$\sigma_{amp}$ [MPa]	$\mathrm{Cycles}\;\mathrm{to}\;\mathrm{Failure},\;N_{f}$
1	44.2	29.5	327,112
2	44.2	29.5	662,683
3	44.2	29.5	369,762
4	47.9	31.9	36,506
5	49.5	32.9	315,418
6	49.5	32.9	70,159
7	39.0	26.0	634,019
8	39.0	26.0	10,000,000

**Table 5:** Fatigue strength comparison with mean stress correction (R=−1).

Ref.	Specimen Type	$\sigma_{u}$ [MPa]	$\sigma_{m}$ [MPa]	$\sigma_{f}$ [MPa]	$\sigma_{f(R=-1)}$ [MPa]
In the present study	3D-printed PEEK	86.7	39.0	26.0	47.3
Avanzini et al. [40]	Extruded PEEK	102.0	40.0	40.0	65.8
Sobieraj et al. [51]	Extruded PEEK—moderate notch	112.0 *	42.0 **	42.0 **	67.1
	Extruded PEEK—razor notch	85.0 *	27.4 **	27.4 **	40.5
Puigoriol-Forcada et al. [56]	3D-printed PC	48.7	4.4 **	1.5 **	1.6
Fischer et al. [57]	3D-printed PEI	72.5 *	6.9 **	6.9 **	7.7
Kiani et al. [61]	3D-printed PLA	61.3	0.0	15.5	15.5
	3D-printed PLA/PCL (80/20)	33.5	0.0	13.6	13.6

* Values estimated from plots. ** Values estimated from the S-N curve fit.

## Data Availability

Data are contained within the article.
